# Relationship between Serum Vitamin D Status and Metabolic Risk Factors among Korean Adults with Prediabetes

**DOI:** 10.1371/journal.pone.0165324

**Published:** 2016-10-26

**Authors:** Han Na Kwon, Hyunjung Lim

**Affiliations:** 1 Department of Medical Nutrition, Graduate School of East-West Medical Science, Kyung Hee University, Yong-In, Gyenggi-do 17104, Republic of Korea; 2 Research Institute of Medical Nutrition, Kyung Hee University, Seoul 02447, Republic of Korea; University of Alabama at Birmingham, UNITED STATES

## Abstract

Serum vitamin D status has been associated with prediabetes and metabolic syndrome. Evidence for the increased risk of metabolic disorders in individuals with prediabetes and a low vitamin D status is limited and uncertain. Furthermore, it has not been confirmed whether this possible relationship occurs in the Korean population. The aim of this study was to assess serum vitamin D status and to examine the relationship between serum vitamin D levels and metabolic risk factors in Korean adults with prediabetes. This cross-sectional study was conducted among 60 subjects aged 20–65 years. Participants had fasting glucose levels of 100 to 125 mg/dl. A questionnaire was used to assess vitamin D synthesis from sun exposure and a dietary intake examined using 3-days dietary records. Clinical and biochemical data were also collected. The 2009 harmonized definition of metabolic syndrome was used. Serum vitamin D levels were classified according to criteria from the 2011 Institute of Medicine report. The majority of subjects (75%) had a serum 25(OH)D level < 20 ng/ml, and among them, 31.1% were vitamin D deficiency (< 12 ng/ml). The proportion (42.9%) of subjects having low HDL-cholesterol was the highest among vitamin D deficiency (< 12 ng/ml) group (12 to < 20 ng/ml: 16.1%, ≥ 20 ng/ml: 6.7%). We observed an inverse relationship between 25(OH)D levels and TG, AI (β = -6.355, SE = 2.463; β = -0.020, SE = 0.008) after adjusted confounders. Korean adults with prediabetes were more likely to have low serum 25(OH)D levels. A sufficient 25(OH)D level may have possible beneficial effects on lipid profiles.

## Introduction

According to data on the Korea National Health and Nutritional Examination Survey, the prevalence of prediabetes, which is one of metabolic syndrome components, has increased steadily to 25.0% among adults aged 30 years and older in 2013 [1,2]. Although several studies have reported beneficial effects of lifestyle modification including nutrition management for preventing of diabetes and metabolic syndrome (MetS) [3,4], these modifications are inadequate to be applied to Korean with prediabetes, owing to a lack of evidences.

Recently, the role of vitamin D in prediabetes has increased interest [5]. Inadequate vitamin D status is highly prevalent in the general population and is now recognized as a common health issue worldwide [6]. In South Korea, it has been shown that there is a high prevalence of poor vitamin D status [7,8]. Prediabetes is defined as an insulin resistance [9]. Accumulating evidences have suggested that vitamin D may have a protective role in the underlying disorders of prediabetes [10,11]. One proposed mechanism of the beneficial effect of vitamin D in prediabetes is that vitamin D directly modulates gene expression in target cells. Both vitamin D receptor and 25(OH)D-1α hydroxylase involved in this mechanism are discovered in most cells and tissues. On that basis, new potentiality of vitamin D have been presented [6].

More recently, there have been an increasing number of studies that have established the relationship between vitamin D deficiency and MetS including its components [11,12]. However, some studies have suggested conflicting results [13,14], and there is still controversy regarding this relationship.

Some U.S studies have suggested the importance of initial management in the prevention of MetS as well as cardio vascular disease (CVD) in prediabetic adults [1,15]. However, to date, no studies have established the relationship between serum vitamin D status and MetS in South Korea. Although there have been several studies among adults with prediabetes or high risk for T2DM [11,14], these results cannot be directly applied to a Korean population due to ethnic differences in vitamin D metabolism and its nutritional status [16].

Therefore, we evaluated serum vitamin D status among Korean adults aged 20 to 65 years with prediabetes (fasting serum glucose levels 100 to 125 mg/dl). We also examined the association between serum vitamin D levels and metabolic risk factors.

## Materials and Methods

### Subjects and study design

This cross-sectional study was approved by the Institutional Review Board at Kyung Hee University Hospital in Seoul, South Korea (KMC IRB 1406–02). Individuals were recruited through a notice on the hospital website and bulletin board. A total of 185 individuals were invited to undergo a screening test for 5 months, from May to October 2014. Individuals aged 20 to 65 years, who had a fasting glucose level of 100 to 125 mg/dl, were eligible for this study. Exclusion criteria included: 1) HbA1c levels 6.5% and over, 2) use of oral hypoglycemic agents, 3) pregnancy, 4) alcoholism, and 5) renal dysfunction or dyshepatia. Based on the exclusion criteria, a total of 118 participants were determined to be ineligible. A total of 7 participants withdrew their consent by oneself.

A 60 subjects (31 male and 29 female) was finally enrolled. All subjects signed a written informed consent. This study was conducted using questionnaires, a dietary intake survey, anthropometric investigation, BP measurement, and blood test.

### Data collection

A general informational survey was used to record self-reported and verified a fact. Medical history, including any diagnosed conditions and diagnosis date, was collected. A drug history survey was used to assess current medication use including drug name, daily dose with unit, period of dose, and purpose of dose. Smoking and drinking alcohol were classified as never, former, or current.

Participants visited in late spring, summer or fall and self-reported one of three degrees of skin color (light, medium, dark), the duration (after washing face, before going out, while one is out) and body part (face, neck, arm, leg, shoulder, whole body) of sunscreen use, as well as sunscreen reapplication habits, and also documented the frequency and mean duration of daytime outdoor activity.

Dietary intake was investigated through 3 days of dietary records. To account for weekly variations in dietary intake, the 3 days of dietary records included one weekend day or holiday. The 3-day dietary records were compared with actual intake using model food during personal interviews. Data from dietary records were analyzed using a computer-aided nutritional analysis program, CAN Pro version 4.0 (The Korean Nutrition Society 2010, Seoul, Korea) to determine subjects’ dietary intake for multiple nutrient categories including vitamin D.

Standing height, weight and body composition were obtained on an empty stomach by bioelectrical impedance analysis (Inbody720, Biospace, Seoul, South Korea). Measurements were recorded to the nearest 0.1cm or 0.1kg. WC was measured horizontally at the navel using a tape measure (Hoechstmass, Sulzbach, Germany). At rest while seated, BP of the upper arms was measured 2 times at 30-seconds intervals using an automatic electronic sphygmomanometer (BPBI0320S, Inbody, Seoul, South Korea) and the average of measurements was used for analysis. To reduce measuring error, the same equipment and instrument was used according to standard methods.

Blood samples were collected in the morning after fasting for 12 hours. After 30 minutes venous blood collection, serum was separated in serum separation tubes by centrifugal filtration (3000rpm for 15minutes), separated serum put into eppendorf tubes using pipeat. Eppendorf tubes and ethylene diamine tetra acetic acid tubes were properly stored, and shipped to the blood analysis facility for analysis. The fasting serum concentration of glucose was analyzed using UV assay (HK). Levels of insulin were analyzed using electro-chemiluminescence immunoassay (ECLIA). Whole blood HbA1c was analyzed using turbidimetric immunoassay (TIA).

Insulin resistance (IR) was approximately estimated by both the Homeostatic Model of Insulin Resistance (HOMA-IR) and Quantitative Insulin Sensitivity Check Index (QUICKI). HOMA-IR was calculated by the following formula:

$$\left(fasting\;insulin\;\left(\frac{mU}{l}\right)\times fasting\;glucose\;\left(\frac{mg}{dl}\right)\right)÷405$$

[17].

The reciprocal of HOMA-IR was used for analysis due to greater reproducibility [18]. QUICKI is a simple, accurate method for evaluating IR that is useful for clinical investigations [19]. It was calculated as the following:

$$1÷\text{log }fasting\;insulin\;\left(\frac{\mu U}{ml}\right)+\text{log }fasting\;glucose\;\left(\frac{mg}{dl}\right)$$

[19].

Serum concentrations of total cholesterol and triglycerides were analyzed using enzymatic colorimetric assay. Levels of high density lipoprotein cholesterol (HDL-cholesterol) and low density lipoprotein cholesterol (LDL- cholesterol) were analyzed using homogeneous enzymatic colorimetric assay. Serum 25(OH)D concentrations were measured using chemiluminescent immunoassay (CLIA). Atherogenic index (AI) was calculated as the following:

(total cholesterol−HDL cholesterol)÷HDL cholesterol

[20].

### Definition of metabolic syndrome

In this study, MetS was defined according to the harmonizing definition from the International Diabetes Federation, the American Heart Association, and the National Heart, Lung, and Blood Institute (AHA/IDF/NHLBI harmonizing definition, 2009) [21].

The presence of 3 or more of the following criteria was used to define MetS:

1. Central obesity, waist circumference (≥ 90 cm for men and ≥ 85 cm for women) [22]
2. Triglycerides (≥ 150 mg/dl) or current use of drug treatment for elevated triglycerides
3. HDL-cholesterol (< 40 mg/dl for men and < 50 mg/dl for women) or current use of drug treatment for reduced HDL-cholesterol
4. Blood pressure (systolic ≥ 130 mmHg or diastolic≥ 85mmHg) or current use of antihypertensive drug treatment
5. Fasting glucose (≥ 100mg/dl) or current use of insulin, oral hypoglycemia drug.

### Diagnostic criteria of vitamin D status

Serum 1, 25(OH)_2_D has a shorter half-life of 4 hours than serum 25(OH)D and should not be used as a measure of vitamin D deficiency. In addition, levels of 1, 25(OH)_2_D may be normal or even elevated as a result of secondary hyperparathyroidism [23]. Therefore, evidence suggests that serum 25(OH)D is the best parameter to assess vitamin D status due to its relatively long half-life of approximately 2–3 weeks [7]. There is a lack of consensus regarding the optimal serum 25(OH)D levels. According to a 2011 report by the Institute of Medicine (IOM), review of the available data suggests that there is a risk for vitamin D deficiency at serum 25(OH)D levels less than 30 nmol/L (12 ng/mL). There is a potential risk for vitamin D inadequacy at serum 25(OH)D levels between 30 and 50 nmol/L (12 and 20 ng/mL). Practically, vitamin D normal occurs at serum 25(OH)D levels of at least 50 nmol/L (20 ng/mL) [24].

### Statistical analysis

Data was presented as mean ± standard error (SE) for numerical data and as numbers and percentage for categorical data. Comparison of general characteristics, mean of metabolic outcomes and proportion of metabolic risk factors by vitamin D status groups including mean of serum 25(OH)D levels of MetS combinations were performed using Fisher’s exact test and non-parametric statistics such as Kruskal-Wallis tests (post hoc by Bonferroni’s method). Partial correlation analysis and multiple linear regression analysis were used to demonstrate a linear relationship between serum 25(OH)D levels and metabolic outcomes. The covariates for the adjusted r and β include age, sex, smoking, drinking alcohol, daytime outdoor activity and energy intake.

All statistical analyses were performed using SPSS (version 21.0, SPSS Inc., Chicago, IL, USA). Statistical significance was defined as a p-value of < 0.05.

## Results

### General characteristics by serum vitamin D status

The general characteristics of Korean prediabetic adults by serum 25(OH)D level are shown in Table 1. Among 60 subjects, 31 (51.7%) were male and 29 (48.3%) were female. The mean age of all subjects was 45.1 ± 1.4 years. Overall, most subjects (75%) had a serum 25(OH)D level < 20 ng/ml, and among these subjects, 31.1% were vitamin D deficient (< 12 ng/ml) according to IOM criteria. The mean serum 25(OH)D level among all subjects was 17.3 ± 0.8 ng/ml.

**Table 1:** General characteristics of Korean prediabetic adults by serum 25(OH)D level.

	Serum 25(OH)D groups by IOM criteria ^a^				
	Total	< 12 ng/ml	12 to < 20 ng/ml	≥ 20 ng/ml	P value
	(n = 60)	(n = 14)	(n = 31)	(n = 15)	
Sex (%)					
Male	31 (51.7) ^b^	5 (35.7)	18 (58.1)	8 (53.3)	0.387
Age (y)	45.1 ± 1.4 ^c^	43.5 ± 3.2	43.7 ± 1.8	49.4 ± 3.1	
Smoking (%)					
Never	40 (66.7)	12 (85.7)	17 (54.8)	17 (73.3)	0.090
Former	2 (3.3)	1 (7.1)	1 (3.2)	0 (0.0)	
Current	18 (30.0)	1 (7.2)	13 (42.0)	4 (26.7)	
Drinking alcohol (%)					
Never	16 (26.7)	5 (35.7)	6 (19.4)	5 (33.3)	0.693
Former	1 (1.7)	0 (0.0)	1 (3.2)	0 (0.0)	
Current	43 (71.7)	9 (64.3)	24 (77.4)	10 (66.7)	
Season of blood draw (%)					
Late spring and summer	24 (40.0)	8 (57.1)	13 (41.9)	3 (20.0)	0.042
Fall	36 (60.0)	6 (42.9)	18 (58.1)	12 (80.0)	
Daytime outdoor activity (%)					
6~7/week	12 (20.0)	1 (7.1)	4 (12.9)	7 (46.7)	0.019
3~5/week	7 (11.7)	3 (21.4)	1 (3.2)	3 (20.0)	
1~2/week	19 (31.7)	5 (35.7)	12 (38.7)	2 (13.3)	
Never	22 (36.7)	5 (35.7)	14 (45.2)	3 (20.0)	
Skin color (%)					
Light	9 (15.0)	4 (28.6)	5 (16.1)	0 (0.0)	0.081
Medium	39 (65.0)	9 (64.3)	17 (54.8)	13 (86.7)	
Dark	12 (20.0)	1 (7.1)	9 (29.0)	2 (13.3)	
Sunblock (%)					
User	24 (40.0)	5 (35.7)	13 (41.9)	6 (40.0)	0.941
Energy intake (kcal/day)	1565.0 ± 63.8	1514.4 ± 76.5	1551.9 ± 108.8	1639.2 ± 103.0	0.363
Total fat intake (g/day)	41.8 ± 2.1	39.3 ± 3.4	40.4 ± 3.2	47.1 ± 3.8	0.196
Vitamin D intake (μg/day)	3.1 ± 0.4	2.9 ± 0.5	2.6 ± 0.3	3.9 ± 1.2	0.741
Vitamin D intake (%, KDRIs) ^d^	30.0 ± 3.7	29.3 ± 4.7	25.8 ± 3.5	39.4 ± 12.3	0.689

25(OH)D, 25-hydroxyvitamin D; IOM, Institute of Medicine

^a^ 25(OH)D levels were classified according to diagnostic criteria defined by IOM in 2011.

^b^ Values are n (%); difference among serum 25(OH)D group was assessed by the Fisher’s exact test.

^c^ Values are mean ± SE; difference among serum 25(OH)D group was assessed by the Kruskal-Wallis test.

^d^ Percentage of daily intake of vitamin D compared with the quantity suggested by dietary reference intakes for Koreans (KDRIs).

The proportion of subjects sampled in the late spring or summer decrease as serum 25(OH)D levels increased (p = 0.042). Approximately one third (36.7%) of subjects reported no outdoor activity during the daytime. The proportion of daytime outdoor activity stayed as much as 6–7 times a week in subjects with vitamin D deficiency (< 12 ng/ml) was the lowest among the three serum 25(OH)D groups (p = 0.019). Skin color and use of sunblock did not differ among serum 25(OH)D levels (all p ≥ 0.05).

The mean daily intake of vitamin D was 3.1 ± 0.4 μg as measured by 3 days of dietary records. These intakes are approximately 30% of the quantity suggested by Dietary Reference Intakes for Koreans (KDRIs).

### Metabolic outcomes by serum vitamin D status

The metabolic outcomes of Korean prediabetic adults by serum 25(OH)D level are shown in Table 2. The mean BMI of all subjects was 25.4 ± 0.5 kg/m^2^. There was significant association between serum 25(OH)D level and atherogenic index (AI) in females only (p = 0.038).

**Table 2:** Metabolic outcomes of Korean prediabetic adults by serum 25(OH)D level.

	Serum 25(OH)D groups by IOM criteria ^a^				
	Total	< 12 ng/ml	12 to < 20 ng/ml	≥ 20 ng/ml	P value ^f^
	(n = 60)	(n = 14)	(n = 31)	(n = 15)	
**Clinical parameters**					
BMI (kg/m^2^)	25.4 ± 0.5 ^b^	25.9 ± 1.3	24.9 ± 0.6	26.0 ± 0.7	0.434
Percent body fat (%)	29.6 ± 0.9	32.8 ± 2.3	28.7 ± 1.0	28.8 ± 2.1	0.238
Waist circumference (cm)					
Male	89.9 ± 1.5	90.4 ± 4.9	90.7 ± 1.8	87.9 ± 2.9	0.871
Female	79.6 ± 1.8	79.5 ± 3.1	78.1 ± 2.2	82.5 ± 5.3	0.888
Systolic blood pressure (mmHg)	122.8 ± 2.1	121.9 ± 3.7	123.9 ± 3.1	121.5 ± 4.0	0.941
Diastolic blood pressure (mmHg)	82.9 ± 1.5	82.5 ± 2.9	83.8 ± 1.8	81.4 ± 3.9	0.752
**Biochemical parameters**					
Fasting glucose (mg/dl)	106.3 ± 0.7	103.8 ± 0.8	106.6 ± 1.0	107.9 ± 1.6	0.160
Fasting insulin (mU/l)	6.2 ± 0.6	7.5 ± 1.8	5.5 ± 0.5	6.5 ± 1.2	0.783
1/HOMA-IR ^c^	1.46 ± 0.31	0.94 ± 0.17	1.45 ± 0.36	1.96 ± 1.02	0.752
QUICKI ^d^	0.38 ± 0.01	0.37 ± 0.01	0.39 ± 0.01	0.39 ± 0.03	0.714
Triglyceride (mg/dl)	148.0 ± 13.9	180.2 ± 31.7	146.9 ± 21.4	119.9 ± 15.1	0.262
Total cholesterol (mg/dl)	205.2 ± 4.9	208.7 ± 10.0	211.1 ± 7.0	189.7 ± 8.6	0.183
LDL-cholesterol (mg/dl)	126.5 ± 4.5	126.9 ± 9.8	132.6 ± 6.5	113.6 ± 7.8	0.230
HDL-cholesterol (mg/dl)					
Male	51.6 ± 2.1	53.2 ± 6.6	50.7 ± 2.5	52.6 ± 5.0	0.961
Female	62.5 ± 3.2	53.4 ± 4.6	63.2 ± 5.4	72.9 ± 4.1	0.077
Atherogenic index ^e^					
Male	0.46 ± 0.06	0.44 ± 0.18	0.50 ± 0.07	0.38 ± 0.11	0.708
Female	0.24 ± 0.06	0.48 ± 0.12	0.14 ± 0.09	0.11 ± 0.06	0.038

25(OH)D, 25-hydroxyvitamin D; IOM, Institute of Medicine; BMI, body mass index; HOMA-IR, homeostatic model assessment of insulin resistance; QUICKI, Quantitative Insulin Sensitivity Check Index; LDL-cholesterol, low density lipoprotein cholesterol; HDL-cholesterol, high density lipoprotein cholesterol.

^a^ 25(OH)D levels were classified according to diagnostic criteria defined by IOM in 2011.

^b^ Values are mean ± SE.

^c^ HOMA-IR = (fasting insulin (mU/L) x fasting glucose (mg/dl))/405.

^d^ QUICKI = 1/log(fasting insulin(μU/mL)) + log(fasting glucose(mg/dl)).

^e^ Atherogenic index (AI) = (total cholesterol–HDL-cholesterol)/HDL-cholesterol.

^f^ Difference among serum 25(OH)D group was assessed by the Kruskal-Wallis test.

### Metabolic risk factors by serum vitamin D status

Table 3 shows the metabolic risk factors of Korean prediabetic adults by serum 25(OH)D level. Approximately 43.3% of all subjects were classified as having MetS. A significant difference in the prevalence of MetS by serum 25(OH)D levels was not found. Low HDL-cholesterol increased as serum 25(OH)D levels decreased (p = 0.047).

**Table 3:** Metabolic risk factors of Korean prediabetic adults by serum 25(OH)D level.

	Serum 25(OH)D groups by IOM criteria ^a^				
	Total	< 12 ng/ml	12 to < 20 ng/ml	≥ 20 ng/ml	P value ^d^
	(n = 60)	(n = 14)	(n = 30)	(n = 15)	
Metabolic syndrome ^c^	26 (43.3) ^b^	7 (50.0)	13 (41.9)	6 (40.0)	0.594
Central obesity	23 (38.3)	5 (35.7)	12 (38.7)	6 (40.0)	0.815
High blood pressure	28 (46.7)	6 (42.9)	15 (48.4)	7 (46.7)	0.844
Hypertriglyceridemia	25 (41.7)	6 (42.9)	13 (41.9)	6 (40.0)	0.876
Low HDL-cholesterol	12 (20.0)	6 (42.9)	5 (16.1)	1 (6.7)	0.047

25(OH)D, 25-hydroxyvitamin D; IOM, Institute of Medicine; HDL-cholesterol, high density lipoprotein cholesterol.

^a^ 25(OH)D levels were classified according to diagnostic criteria defined by IOM in 2011.

^b^ Values are n (%).

^c^ Metabolic syndrome was Defined using the harmonizing definition of IDF and AHA/NHlBI in 2009.

^d^ Difference among serum 25(OH)D group was assessed by the Fisher’s exact test.

### Serum 25(OH)D levels according to the combinations of metabolic risk factors

**Fig 1:**
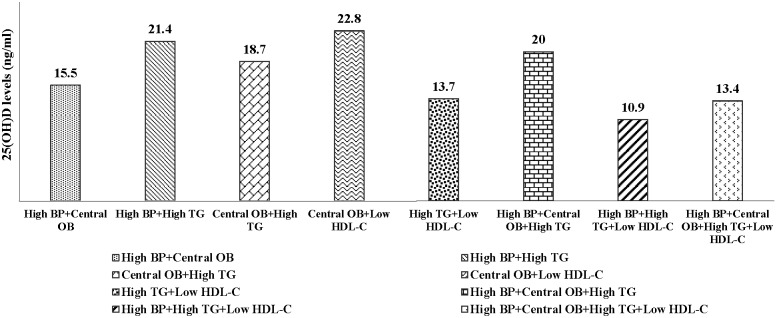
Comparison of mean serum 25(OH)D levels of MetS combinations. 25(OH)D, 25-hydroxyvitam in D; MetS, metabolic syndrome; BP, blood pressure; OB, obesity; TG, triglyceride; HDL-C, high density lipoprotein cholesterol. Comparison of mean serum 25(OH)D levels of subjects with various MetS combinations. NS: Non Significantly difference by serum 25(OH)D levels for the kruskal-wallis test.

Comparison of mean serum 25(OH)D levels according to MetS combinations among prediabetic adults are shown in Fig 1. There were no subjects with combinations of (1) high blood pressure, low HDL-cholesterol (High BP+Low HDL-C), (2) high blood pressure, low HDL-cholesterol, central obesity (High BP+low HDL-C +Central OB) and (3) central obesity, hypertriglyceridemia, low HDL-cholesterol (central OB+High TG+Low HDL-C). Subjects with combination of high blood pressure, hypertriglyceridemia, low HDL-cholesterol (High BP+High TG+Low HDL-C) had the lowest serum 25(OH)D levels (10.9 ng/ml). Mean 25(OH)D levels did not differ by MetS combinations.

### Linear relationship between serum 25(OH)D levels and metabolic outcomes

The results of the linear regression analysis to identify the relationship between serum 25(OH)D levels and metabolic outcomes are shown in Table 4. Serum 25(OH)D levels were inversely associated with TG (β = -6.355) and AI (β = -0.020) after adjustment for age, sex, smoking, drinking alcohol, daytime outdoor activity and total energy intake (TG, p = 0.013; AI, p = 0.010, respectively). S1 Table shows the linear relationship between serum 25(OH)D levels and 1/HOMA-IR and QUICKI. Serum 25(OH)D levels showed a significantly positive correlation to 1/HOMA-IR (r = 0.389) and QUICKI (r = 0.306) after adjustment for age, sex, smoking, drinking alcohol, daytime outdoor activity, and total energy intake (1/HOMA-IR, p = 0.007; QUICKI, p = 0.022, respectively).

**Table 4:** Linear regression coefficients between serum 25(OH)D level and metabolic outcomes among Korean prediabetic adults.

	Unadjusted		Adjusted ^b^	
	β (SE)	P value	β (SE)	P value
Waist circumference (cm)	0.254 (0.226)	0.266	0.062 (0.230)	0.787
Systolic blood pressure (mmHg)	0.492 (0.349)	0.164	0.175 (0.363)	0.632
Diastolic blood pressure (mmHg)	0.348 (0.252)	0.173	0.305 (0.264)	0.254
Triglyceride (mg/dl)	-3.510 (2.339)	0.139	-6.355 (2.463)	0.013
HDL-cholesterol (mg/dl)	0.431 (0.341)	0.212	0.738 (0.389)	0.064
Atherogenic index ^a^	-0.010 (0.007)	0.199	-0.020 (0.008)	0.010

25(OH)D, 25-hydroxyvitamin D; HDL-cholesterol, high density lipoprotein cholesterol.

^a^ Atherogenic index (AI) = (total cholesterol–HDL-cholesterol)/HDL-cholesterol.

^b^ adjusted for age, sex, smoking (never, former, current), drinking alcohol (never, former, current), daytime outdoor activity (never, 1–2, 3–5, 6–7) and total energy intake.

## Discussion

This study found that three fourths (75%) of Korean prediabetic adults had a low serum 25(OH)D levels. Although significant association between a low serum vitamin D status and MetS including central obesity and high blood pressure did not find, vitamin D deficient subjects among prediabetic Korean adults were exposed to an increased TG levels and had an elevated risk of arteriosclerosis.

In this study, serum 25(OH)D levels showed a significantly positive correlation to 1/HOMA-IR and QUICKI after adjusted confounders. We did not find a relationship between serum 25(OH)D levels and 1/HOMA-IR, QUICKI by the multiple linear regression analysis in contrast with the results from the majority of studies [10, 11, 24]. Currently, there was a lot of studies to examine whether a causal relationship exists between vitamin D supplementation and the progression to T2DM in individual with prediabetes [25,26]. There are multiple potential pathways underlying the relationship between vitamin D and glucose metabolism [27–30].

Shin et al. [31] suggested that impaired lipid profiles were commonly associated with CVD and can also occur in individual with prediabetes. Lauer et al. [20] presented that individual with low HDL-cholesterol had a higher risk of CVD events irrespective of LDL-cholesterol and AI made an accurate estimate ischemic heart disease events.

We observed that TG and AI decreased per 1ng/ml increase in serum 25(OH)D levels after adjusted confounders. Among vitamin D deficiency (< 12 ng/ml) group, the proportion (42.9%) of subjects with low HDL- cholesterol was higher than insufficiency level (12 to < 20 ng/ml, 16.1%) and normal vitamin D level (≥ 20 ng/ml, 6.7%). The major finding of our study was an association between vitamin D and lipid profiles.

Mostly, our results was consistent with those of the studies among individuals having conditions related IR, adiposity and inflammation such as prediabetes, high risk of T2DM, obese, old age and menopause. For example, a cross-sectional study [11] among 390 Canadian adults (aged median 34 years) with a high risk for T2DM and mean BMI 29.8 kg/m^2^, showed that TG decreased as much as 0.14 mmol/l per 1nmol/l increase in serum 25(OH)D levels. In another study [32] of 73 European descent adults being morbidly obese (BMI ≥ 40kg/m^2^), serum 25(OH)D levels were negatively correlated with TG (r = -0.364). Chon et al. [33] suggested that Korean postmenopausal women in the highest serum 25(OH)D level showed a significant decrease in the prevalence of hypertriglyceridemia (ORs = 0.83, 95% CI = 0.71–0.97) and low HDL-cholesterol (ORs = 0.80, 95% CI = 0.69–0.93). Also, Lu et al. [12] reported that among 3262 Chinese individuals aged 50–70 years, TG decreased as much as 0.10 mmol/l and HDL-cholesterol increased as much as 0.06 mmo/l per 1nmol/l increase in serum 25(OH)D levels.

In contrast to results of studies already presented, a cross-sectional study [34] among 355 non-diabetic young adults (aged mean 23.5 years) with a mean BMI 23.8 kg/m^2^, showed that there was no significant correlation between serum 25(OH)D levels and lipid profiles such as TG and HDL-cholesterol.

Although the mechanistic impact of vitamin D on lipid profiles is not clear, several mechanisms [35,36] have been proposed: vitamin D may increase lipoprotein lipase gene expression, increasing removal of lipoprotein particles; or, hyperparathyroidism due to low 25(OH)D levels may decrease peripheral removal of TG and contribute to activation of microsomal triglyceride transfer protein by hepatocellular Ca+, which is able to induce hypertriglyceridemia. A study [37] has suggested that vitamin D may regulate macrophage function on reverse cholesterol transport and large HDL particles increased by taking over cholesterol from macrophage. In addition, Zhou et al. [38] proposed that vitamin D may improve free fatty acids-induced IR. Kang et al. [39] suggested that vitamin D may stimulate a gene expression of cytokine in macrophage and perform many systemic anti-inflammatory actions. Most recently, Slominski et al. [40] reported that novel vitamin D_3_-hydroxyderivatives are noncalcemic unlike 1, 25(OH)_2_D_3_ and may promote anti-inflammatory activity. Thereby Guasch et al. [41] explained vitamin D indirectly influence lipid metabolism mediated by IR and inflammation.

This evidences discussed earlier may provide support for relationship between vitamin D and TG, HDL-cholesterol, or AI among individuals having conditions related IR, adiposity, and inflammation.

Although Korea is located at latitudes 33–38°N, which does allow for adequate ultraviolet B for vitamin D synthesis, Korea has one of the highest prevalence of vitamin D deficiency [7]. Previous studies [33,42] suggest that 56.0–62.1% of Korean adults are vitamin D insufficient (25[OH]D <20ng/ml). Our study found that the prevalence of 25(OH)D <20ng/ml among adults with prediabetes was higher than in previous studies at 75% of all adults. Gupta et al. [9] and Abbasi et al. [10] suggested that mean 25(OH)D levels are lower in individuals with prediabetes, than in those who are normoglycemia. This study found that approximately one in three prediabetic adults in Korea did not engage in outdoor activity during the daytime and the mean of vitamin D intake was 3.1 ± 0.4 μg/day. Based on this results, the high prevalence of vitamin D deficiency among Korean prediabetic adults could be explained by an indoor lifestyle, low consumption of both vitamin D rich foods and vitamin D fortified foods. [43].

Limitations to our study were the small sample size and cross-sectional design, which does not prove a causal relationship. We did not take into consideration parathyroid hormone (PTH) levels, inflammatory markers, and menopausal status which are possible intermediate confounders. Although we performed a stratified analysis by age at menopause (50 years old), there was no significant relationship. Despite these limitations, to our knowledge, this is the first study to investigate the relationship between serum vitamin D status and metabolic risk factors among Korean adults with prediabetes. Sunlight exposure and vitamin D intake were measured and we could verify difference of vitamin D source by serum vitamin D status. In addition, our study considered whether confounders interact with metabolic risk factors.

In conclusion, Korean prediabetic adults generally had a low vitamin D status. Korean adults having both prediabetes and vitamin D deficiency may be interestingly more accompanied with low HDL-cholesterol. Vitamin D may be beneficial role on dyslipidemia, high TG level induced from prediabetes and reduce the risk of atherosclerosis. However, there was no relationship between vitamin D deficiency and MetS including central obesity and high blood pressure. Further longitudinal or intervention studies are needed in order to investigate whether vitamin D may play a role in the prevention of dyslipidemia, MetS, eventually along with CVD among prediabetic adults.

## Supporting Information

S1 Table Partial correlation coefficients between 25(OH) level and 1/HOMA-IR, QUICKI among Korean prediabetic adults. (XLSX)
